# Assessment of Capacity to Capture DNA Aerosols by Clean Filters for Molecular Biology Experiments

**DOI:** 10.1264/jsme2.ME18012

**Published:** 2018-06-16

**Authors:** Yuki Morono, Tatsuhiko Hoshino, Takeshi Terada, Taketo Suzuki, Takahiro Sato, Hisashi Yuasa, Yuji Kubota, Fumio Inagaki

**Affiliations:** 1 Geomicrobiology Group, Kochi Institute for Core Sample Research, Japan Agency for Marine-Earth Science and Technology (JAMSTEC) Monobe B200, Nankoku, Kochi 783–8502 Japan; 2 Geobiotechnology Group, Research and Development Center for Submarine Resources, JAMSTEC Monobe B200, Nankoku, Kochi 783–8502 Japan; 3 Marine Works Japan Ltd. Oppamahigashi 3–54–1, Yokosuka 237–0063 Japan; 4 Koken Ltd. 7 Yonbancho, Chiyoda-ku, Tokyo 102–8459 Japan; 5 Research and Development Center for Ocean Drilling Science, JAMSTEC 3173–25, Showa-machi, Kanazawa-ku, Yokohama city, Kanagawa 236–0001 Japan

**Keywords:** DNA contamination, ultra-sensitive molecular approach, experimental quality control

## Abstract

Experimental contamination by exogenous DNA is a major issue in molecular biological studies for data quality and its management. We herein assessed DNA aerosols for the risk of contamination and tested the capacity of clean air filters to trap and remove DNA aerosols. DNA aerosols were generated by atomizing a DNA solution and introduced into a laminar flow clean air unit. Capture and detection performed upstream and downstream of the clean air unit showed that a significant fraction (>99.96%) of introduced molecules was trapped and removed by the filter. Although DNA aerosols appear to be an avoidable source of exogenous contamination, a clearer understanding and careful experimental procedures are needed in order to perform contamination-free, high-quality molecular biology experiments.

In parallel to the development of molecular biology techniques that enable the ultra-sensitive detection of DNA/RNA molecules (6, 15, 16), concerns regarding experimental contamination by exogenous molecules in experimental environments, which leads to false-positive results, are increasing (2). The preparation of reaction mixtures is often conducted in a HEPA filter-equipped PCR bench in order to avoid contamination from exogenous sources of DNA. However, in the routine amplification of the same or similar target sequences (*e.g.*, the 16S rRNA gene), PCR amplification may occur in the absence of a template control reaction, potentially reducing experimental quality. Although various measures have been developed in order to avoid contamination from various sources (3–5, 9–12, 14, 17–19), DNA in the form of aerosols (floating particles in the air) in laboratory environments produced through frequent molecular biology experiments (14) have rarely been assessed as a possible source of contamination. In the present study, we conducted model experiments in which various concentrations of DNA aerosols as artificial contaminants were loaded into a laminar flow clean air generator, and the potential of DNA aerosols to cause contamination in a clean air environment was then objectively evaluated. The results obtained are useful for assessing contamination sources and establishing ideal laboratory experimental conditions for contamination-free, highly sensitive molecular biology experiments.

## Materials and Methods

### Preparation of DNA fragments as an artificial contamination source

As a model contaminant DNA, we prepared a 160-bp fragment of Lambda DNA by PCR using Prime STAR MAX (Takara Bio, Otsu, Japan) with 10 pg of Lambda DNA (Takara Bio) as a template and 300 nM each of the primers LAM-F2 (gctgcatactgatgcacttcacg) and LAM-R (ggttatcgaaatcagccacagcg). The amplified product was purified by excising the corresponding size of the band on an agarose gel after electrophoresis and purified by a spin column (Nucleospin Gel and PCR Clean-up, Takara Bio). Concentrations were measured fluorometrically using a Quant-iT PicoGreen dsDNA Assay Kit (Thermo Fisher Scientific, Waltham, MA, USA).

### Experimental set-up

**Fig. 1:**
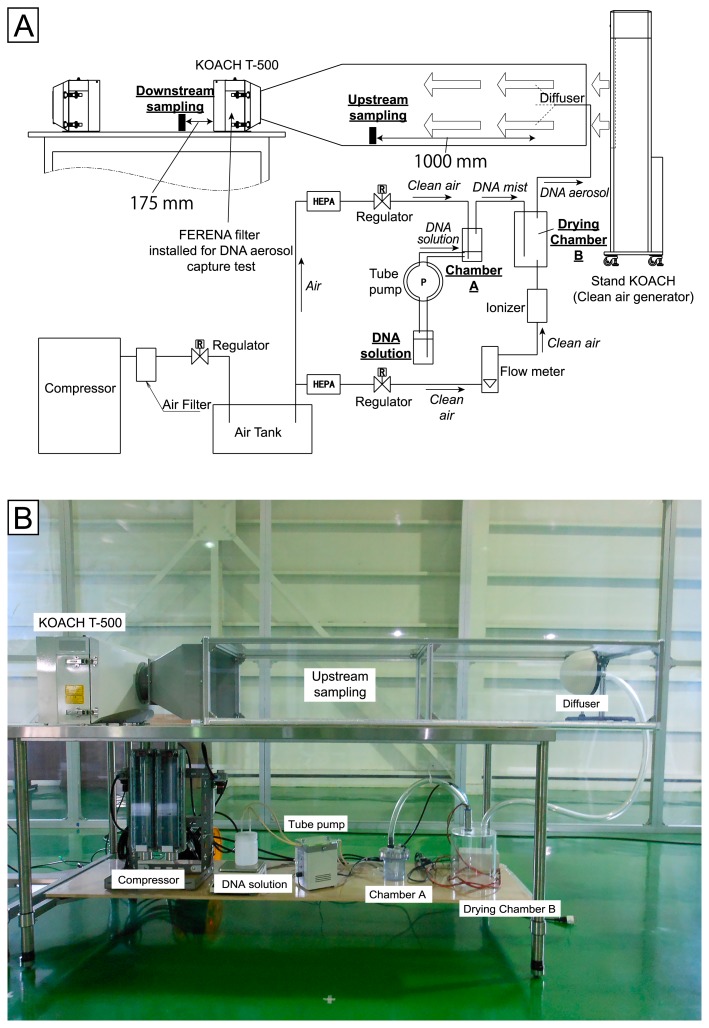
Experimental set-up. Schematic diagram (A) and a picture (B) are shown.

The experimental set-up is shown in Fig. 1. Throughout the experiment, we used a KOACH portable laminar flow clean air system (Localized Air Purifier System, Kubota, Y., P5127292, and Clean air blowing unit, Nitta, K., Fujisiro, Y., Fukiura, K., Sato, T., P5484516, Japan Patent Office) that produces laminar flow air through the clean filter FERENA (Super High-Performance Air filter; Fukazawa, Y. and Kimura, K., P4902788, Japan Patent Office).

Fragments (160 bp) of Lambda DNA were dissolved in water at a concentration of 5.0×10^5^ to 2.5×10^11^ copies mL^−1^, and atomized by aeration with clean air from a compressor in atomizing chamber A (Ultrapure MilliQ water was used for negative control experiments). A fine mist of the DNA solution was then introduced into drying chamber B, in which the mist was dried and turned into DNA aerosols by mixing with dry and ionized air (70 L min^−1^). DNA aerosols were then transferred to the diffuser and flowed into the experimental chamber. In order to minimize any interference by airborne particles, clean air from a Stand KOACH unit (KOACH C 900-F; Koken, Tokyo, Japan) was used as the input air to be mixed with the DNA aerosols produced. DNA aerosol-containing air was then introduced into the Table KOACH (KOACH T 500-F; Koken) unit with a FERENA filter in order to assess its capacity to trap and remove DNA aerosols. Prior to the test, we evaluated the face velocity of air flow up- and downstream using a multi-point anemometer (Kanomax Japan, Osaka, Japan). The cross-sectional area upstream of the chamber matched that downstream, and face velocity was 0.40±0.03 (average±standard deviation) m s^−1^. The flux of DNA molecules was calculated from the volume of loss and concentration of Lambda DNA solution in atomizing chamber A and the cross-sectional area of the exposure unit (1,550 cm^2^). Since the filter potentially accumulates DNA aerosols upon filtration, we avoided repeated experiments in order to avoid any effect of the preceding experiment. Therefore, a higher concentration of aerosol DNA was applied in order for any remaining captured DNA from the preceding experiment to be negligible relative to that applied.

### Monitoring particles, aerosols, and DNA molecules

Since the expected size of the DNA aerosol was below the detection limit of the ordinal particle counter, the size distribution of nanometer-sized particles in the experimental environment was monitored with a Scanning Mobility Particle Sizer Spectorometer (Model 3936; TSI, Shoreview, MN, USA). In order to measure and compare the filtration capacities of particles, we measured particle concentrations upstream (C_up_) and downstream (C_down_) using the Condensation Particle Counter 3022A (TSI). A HEPA filter (Model 3801-6X; Nitta, Osaka, Japan) was installed in the Table KOACH unit in order to measure its filtration capacity. Filtration capacity (%) was assessed using the following equation:

$$\text{Filtration capacity }(\%)=100-100({\text{C}}_{\text{down}}/{\text{C}}_{\text{up}})$$

In the molecular detection of DNA aerosols up- and downstream of the Table KOACH unit, 384 well PCR plates were each placed vertically for 30 min to capture DNA aerosols. The DNA molecules captured were detected by qPCR (ViiA 7 Real-Time PCR System; Thermo Fisher Scientific) using the KAPA SYBR^®^ FAST ROX™ Low Kit (Kapa Biosystems, Wilmington, MA, USA) with the primer pair LAM-F2 and LAM-R shown above for detecting Lambda DNA fragments.

## Results and Discussion

### Production of DNA aerosols

The linearity of DNA aerosol production was confirmed by the loss of the DNA solution in atomizing chamber A (Fig. S1) at a bubbling air pressure range of 0.1 to 0.3 MPa. We used a bubbling air pressure of 0.2 MPa for atomization, which resulted in a mist production rate of 0.253 mL min^−1^ throughout experiments. The number of DNA molecules that virtually passed through the area of 384 well plates (99.6 cm^2^) was calculated by the area ratio and flux of DNA molecules and shown in “Theoretical number of DNA molecules exposed” in Table 2.

### Monitoring particulate matter

**Fig. 2:**
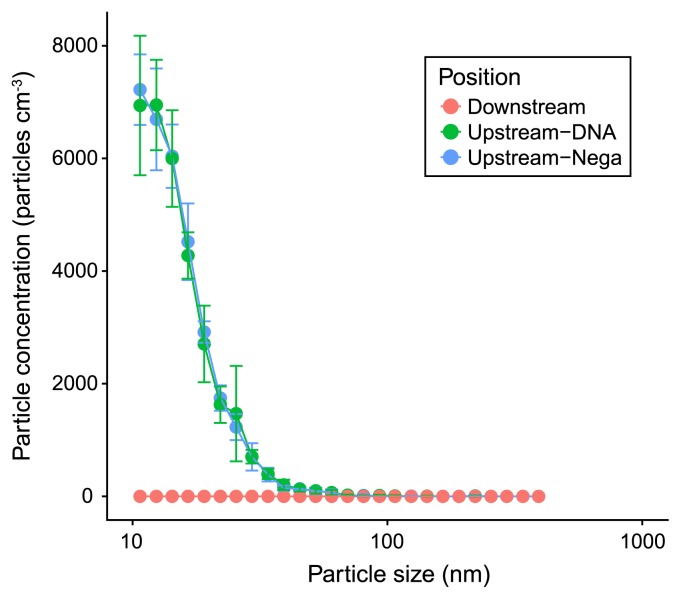
Monitoring results of particulate matter up- and downstream of the filter. Regarding upstream measurements, particulate matter was monitored for DNA solution (5.0×10^6^ copies mL^−1^, Upstream-DNA) or ultrapure MilliQ water (Upstream-Nega) in atomizing chamber A. Error bars show the standard deviation of six measurements.

The size and concentration of particulate matter in the air with and without DNA aerosols, and also those in air downstream of the clean air filter were measured. The results obtained showed that even though ultrapure MilliQ water was used as a negative control, small but measurable particles were produced by the aerosol production units used in the present study (Fig. 2). Measurable particles, which were 10–100 nm in size, were detected and showed an indistinguishable size distribution regardless of the presence or absence of DNA aerosols in the solution in the atomizing unit. The size of DNA aerosols produced by 160-bp DNA fragments in the present study was estimated to be approximately 6 nm (according to the direct measurement results reported by Mouradian *et al.* [13]), which was below the detection range of the particle sizer employed. Two possible interpretations may explain the absence of differences in the distributions of particle sizes measured for aerosols (upstream) with and without DNA shown in Fig. 2: (1) the size of DNA aerosols was not detectable by the particle sizer used in the present study, and (2) the number of DNA aerosols was too small to affect the size distribution of aerosols. In both cases, most of the 10–100-nm-sized particles detected were most likely generated from pure water as a form of particles from impurities. Conclusively, these particles were effectively trapped and removed by the filter installed in the Table KOACH system (Fig. 2, Downstream).

**Fig. 3:**
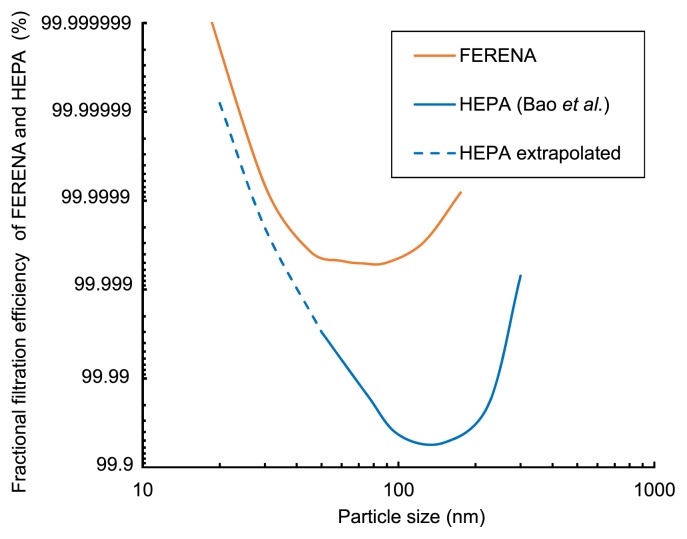
Particle size-dependent filtration efficiencies of FERENA and HEPA filters. Data were obtained from KOKEN Co. Ltd for the FERENA filter (http://www.koken-ltd.co.jp/english/product/clean/super/basis.html) and from Bao *et al.* (1) for the HEPA filter. The particle size range of 10–40 nm for the HEPA filter was estimated by extrapolating from original data.

In the present study, we used the FERENA filter-equipped Table KOACH system, which has a finer mesh structure than the HEPA filter (detailed information is found at http://www.koken-ltd.co.jp/english/product/clean/super/basis.html). The filtration efficiency of aerosol particles produced in the present study for the FERENA filter was measured and compared with the HEPA filter as the percentage of particles trapped by these filters (Table 1). Although a difference was observed in the filtration efficiencies of HEPA (1) and FERENA (Fig. 3), the size distribution of the aerosols produced in the present study (Fig. 2) was such that both filters exhibited similar filtration efficiencies. These results suggest that most of the nanometer-sized aerosols were effectively and similarly captured by both of the clean air filters.

### Molecular detection of DNA aerosols

DNA aerosols were captured on 384 well PCR plates and then quantified by the qPCR reaction (Table 2). The number of DNA molecules detected upstream of the filter ranged between 2.4 and 30.7% of a theoretical number of DNA molecules exposed to the 384 well plates. Under experimental conditions in which higher concentrations of DNA solutions were placed in atomizing chamber A (conditions 2 and 3), the number of molecules detected linearly correlated with the theoretical number of DNA molecules that virtually passed through the area of the 384 well plates, and the percentages of detected/theoretical molecules were similar under these conditions. However, the number of PCR-positive wells did not show a proportional increase, but exhibited an elevated fractional power function to the theoretical number of DNA molecules in the aerosol. If DNA aerosols are composed of a single or few molecule(s), the number of PCR-positive wells is expected to linearly increase as the theoretical number of DNA molecules becomes higher, thereby producing a larger number of DNA aerosols in the air flow, and, thus, the random capturing of aerosols is expected to result in an increase in the number of PCR-positive wells. However, the results obtained, together with those showing an increase in the number of DNA molecules detected per well, indicated that DNA aerosols are an aggregate of a larger number of DNA molecules under the conditions employed in this experiment.

The percentages of captured DNA molecules against the theoretical numbers of DNA molecules (30.7 and 27.9% for conditions 2 and 3, respectively) were consistent with the relative surface area of the wells (30.4%) in the total area of 384 well plates from a quantitative standpoint. This result suggested that DNA aerosols were effectively captured with in sufficient quantity for our following discussion on DNA aerosol filtration by clean filters.

Downstream of the filter, the detection of DNA molecules was markedly reduced and only occurred at the highest exposure condition of DNA aerosols (*i.e.*, condition 3). The number of detected molecules downstream of the filter was 0.039% of the molecules detected upstream. This result showed that more than 99.96% of DNA aerosols were trapped and removed by the FERENA filter; however, this value was lower than the FERENA filter capacity to capture at least 99.9998% of particles with a diameter of 0.15 μm (http://www.koken-ltd.co.jp/english/product/clean/super/basis.html). According to the theory of filtration mechanisms described by Hinds (7), all filters have an intrinsic particle size range that gives minimum efficiency at approximately 50–500 nm. In the case of FERENA filters, it is 50–80 nm (Fig. 3). Particulate matter outside that size range (smaller or larger) are deposited onto the filter fiber and efficiently removed from the air. The size range of DNA aerosols calculated by the equation described by Mouradian *et al.* (13) (Fig. S2) showed that DNA aerosols with 500–2,000 copies of 160 bp-DNA fragments are in the range of 50–80 nm, which is the minimum efficiency for filtration by the FERENA filter. The numbers of detected DNA molecules in two wells in the condition 3 experiment were 523 and 63 copies. The detection of >500 copies of DNA per well supports the hypothesis, and 63 copies, of which the estimated size (24–26 nm) was in the range for efficient removal by the filter, implies the potential disintegration of the aerosol during or after filtration. However, since these results are from only two observations, statistical reliability is low.

Based on the assumption that DNA aerosols exist as an aggregate of 500–2,000 copies of the 160-bp fragment of Lambda DNA, we calculated the contribution of DNA aerosols to the total aerosol shown in Fig. 2. Under conditions 2 and 3 in Table 2, the estimated numbers of the aerosol (with 500–2,000 copies of DNA molecules) were 7–30 and 760–3,039 per 30-min exposure, respectively. Since the flow rate of the Table KOACH unit is 3.83 m^3^ min^−1^, the total volume of air that passed through the area of 384 well plates was calculated to be 2.46×10^5^ cm^3^. Thus, the number of DNA aerosols cm^−3^ of air was expected to be in the range of 0.003–0.012 aerosols, which is markedly lower than the number of aerosols produced from ultrapure water (Fig. 2a). This result appears to explain the absence of a difference in the size distribution of particulate matter with and without DNA in the solution in atomizing chamber A.

### Concluding remarks: Are DNA aerosols a serious contamination source for molecular biology experiments conducted under clean air conditions?

The results of the present study indicate that contamination by DNA aerosols may be present in rare cases under clean air conditions. Even with the active bubbling of 5×10^6^ copies mL^−1^ of DNA solution, the number of aerosols formed was calculated to be up to thousands for a 30-min exposure. The air filter examined in the present study efficiently trapped and removed artificially produced DNA aerosols at a percentage of 99.96%. These results demonstrated that the number of aerosols formed in general molecular biology experiments is small and contamination may be avoided by careful, but basic procedures under laminar flow clean air conditions. However, even though a single DNA molecule has a diameter that is effectively captured by the air filter, extreme care is needed when using high concentrations of DNA solutions in the laboratory, such as PCR products or plasmid solution that may produce aerosols with a larger number of molecules. Since air filters have an intrinsic particle size range that gives minimum efficiency, aerosols with thousands of DNA molecules may pass through air filters and cause contamination.

Our results also indicated that the DNA aerosol itself was trapped and removed by the HEPA filter. However, there can be different situations for the DNA molecules bound to other airborne particles. In the present study, all experiments were performed under the extremely clean air condition of ISO class 1, which contains smaller than ten of 0.1-μm-sized particles in m^−3^ of air (8) produced by the Table KOACH or Stand KOACH system. In the case of a clean bench with ISO class 5 air quality, 10^5^ of 0.1-μm-sized particles, and 10^4^ of 0.3-μm-sized particles are present in 1 m^3^ of air. To the best of our knowledge, the adsorption of DNA onto these airborne particles has not been examined in detail; however, if these particles adsorb DNA and present as larger particle-bound DNA inside a clean bench, they may be the source of contamination even when small-sized DNA aerosols are efficiently trapped by the filter for a clean experimental environment.

## Supplementary Material



## Figures and Tables

**Table 1:** Filtration capacity comparison between FERENA and HEPA filters measured as the percentage of particles captured by these filters

	Average particle concentration* upstream of the filter (Particles cm^−3^)	Average particle concentration* downstream of the filter (Particles cm^−3^)	Filtration capacity (%)
HEPA	1.09×10^4^	0.087	99.9992
FERENA	1.14×10^4^	<0.01	>99.9999

* Particles larger than 7 nm were collectively measured for calculating concentrations

**Table 2:** Summary of experimental conditions and detection results of DNA in aerosols

Experimental conditions	DNA concentration in the solution in chamber A (copies mL^−1^)	Volume of atomized water or DNA solution (mL)	Total number of DNA molecules in the aerosol (copies)	Theoretical number of DNA molecules exposed to 384 well plates	Upstream				Downstream		
	
					Number of PCR-positive wells	Number of detected molecules (copies)	Percentage of detected/ theoretical molecules (%)	Average number of molecules per well (copies)	Number of PCRpositive wells	Number of detected molecules (copies)	Average of molecules per well (copies)
Negative control	0	12.00	0	0	0	0	—	0	0	0	0
Condition 1	5.0×10^2^	16.96	8.48×10^3^	5.45×10^2^	1	13	2.4	13	0	0	0
Condition 2	5.0×10^4^	15.35	7.68×10^5^	4.93×10^4^	18	1.51×10^4^	30.7	(8.41±4.05)×10^2^	0	0	0
Condition 3	5.0×10^6^	16.97	8.49×10^6^	5.45×10^6^	288	1.52×10^6^	27.9	(5.27±3.05)×10^3^	2	5.87×10^2^	293
