# Supplementary material for: Assessment of Capacity to Capture DNA Aerosols by Clean Filters for Molecular Biology Experiments
Source: Microbes Environ. 2018 Jun 16;33(2):222–6. doi: 10.1264/jsme2.ME18012 (PMC6031387; doi:10.1264/jsme2.ME18012)
Supplement: Supplementary file 1 [file 33_222_s1.pdf]

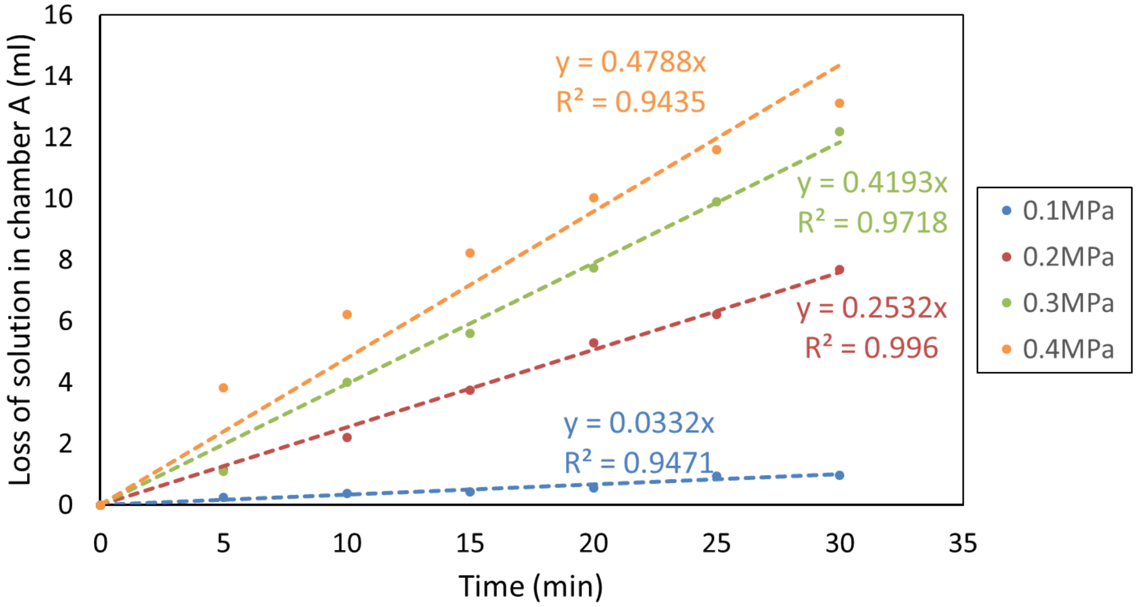


**Fig. S1.** Loss of DNA solution in atomizing chamber A. The loss of water in the atomizing chamber A were monitored with different pressure of air applied for bubbling.

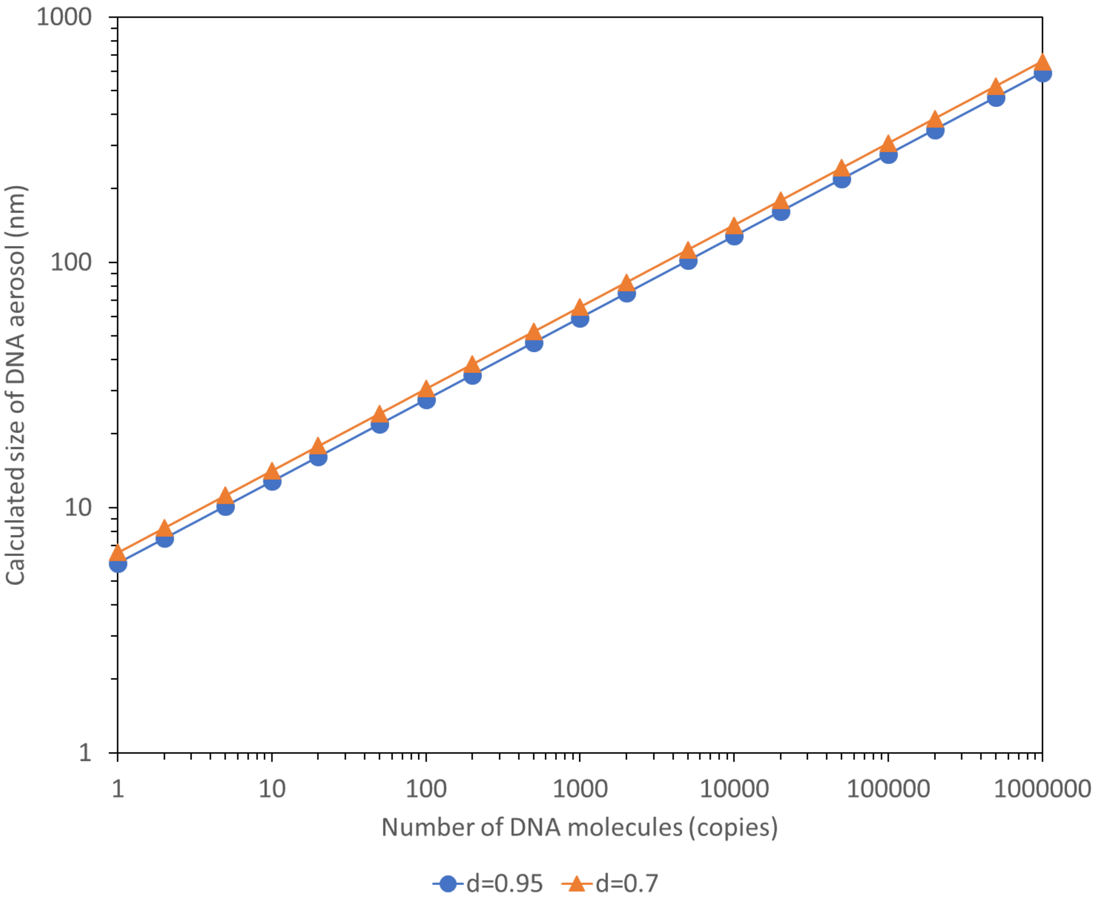


**Fig. S2.** Simulated size range of DNA aerosol based on the equation described by Mouradian et al. The calculation was conducted for two different density (g $cm^{-3}$) assumption for DNA aerosols according to Mouradian et al.

**Reference**

Mouradian S, Skogen JW, Dorman FD, Zarrin F, Kaufman SL, Smith LM. 1997. DNA Analysis Using an Electrospray Scanning Mobility Particle Sizer. Analytical Chemistry 69:919-925.
